# 

**Published:** 1895-09

**Authors:** 


					﻿Vol. XV.
SEPTEMBER, 1895.
NO. 9/’
THE
pOMEOPATHlC pHY^lClAJfl,
A Monthly Journal of Homoeopathic Materia Medica
and Clinical Medicine.
“ He is a freeman whom truth makes free, and all are slaves beside."
“Seek the Truth: come whence it may, cost what it will."
EDITED AND FCBI.I8HKD BY
WALTER M. JAMES, M. D.
PHILADELPHIA :
TSTo. 1231 LOCUST STREET.
& o yr 3D o w :
ALFRED HEATH & CO.; Sole Agents for Great Britain,
114 Ebury Street, Eaton Square.
2^ Yearly Subscription, $2.50, invariably in advance. Single numbers, 25 els.
Entered at the Philadelphia Post-Office as Second-Class Mall Matter.
				

